# Unusual Spinal Dysraphic Lesions

**DOI:** 10.1155/2013/210301

**Published:** 2013-09-30

**Authors:** Jose Roberto Tude Melo, Pollyana Pacheco, Luiz Eduardo Wanderley

**Affiliations:** Pediatric Neurosurgical Unit, Hospital Pediátrico Martagão Gesteira, Rua Jose Duarte 114, Tororó, 40000 Salvador, BA, Brazil

## Abstract

Human tail and multiple spinal dysraphism are unusual congenital malformations. Human tail appeared as a prominent lesion from the lumbosacrococcygeal region, generally without connection between the tail and the neurospinal axis. Spinal dysraphisms are usually isolated, reaching 0.038% of incidence of multiple spinal dysraphisms in the same child. There were three cases described of unusual spinal dysraphic lesions: two cases of human tail and a case of a multiple thoracic myelomeningocele. The literature about diagnosis and treatment was reviewed. Microsurgical technique was performed to provide better exploration of the lesions, and resection could be done in those congenital malformations, without morbidity.

## 1. Introduction 

The neural tube closure occurs through intersection of waves (at least five) which comes from different spots of the craniospinal axis [1]. The human tail is a rare congenital dysraphic anomaly, with prominent lesion from the lumbosacrococcygeal region [2] and, sometimes, with intradural components, that is why the magnetic resonance image (MRI) is mandatory during diagnosis investigation [3]. Regarding myelomeningocele (MM), the literature about the subject assumes that it is formed by a compromising of the union between the fourth and fifth wave [1], usually isolated, being of 0.038% the incidence of multiples spinal dysraphism [4]. Three unusual cases of spinal dysraphism are described here: two infantile cases of human tail and a newborn with a multiple thoracic MM. The families and caregivers have agreed and authorized the publishing of all cases. 

## 2. Case 1

**Figure 1:**
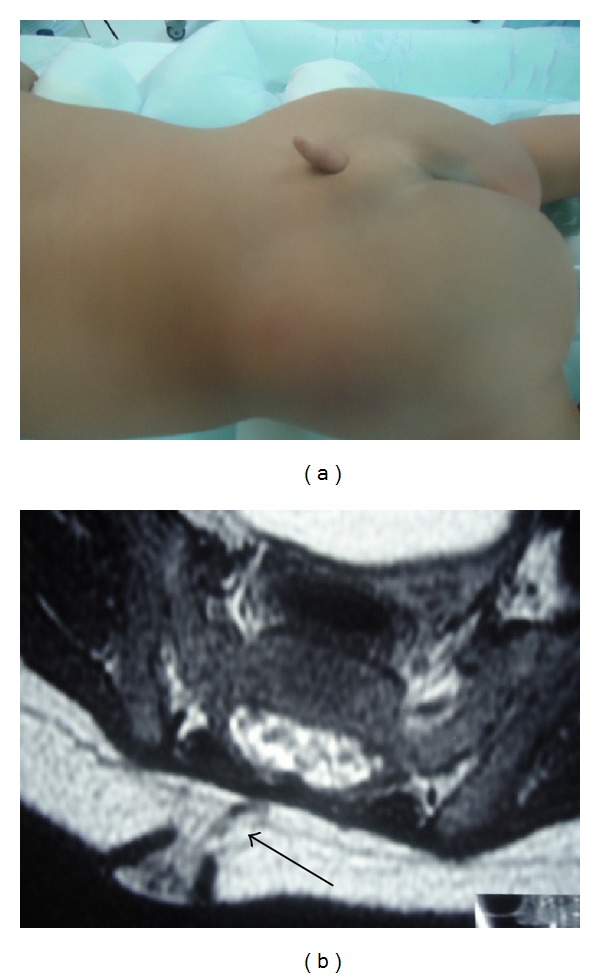
(a) An appendage lesion with approximately 2 cm with no communication with the spinal cord. (b) Confirmation by magnetic resonance image (axial image), with development of a false human tail.

A female child was born with a prominent lesion from the lumbar region, without any kind of neurological disabilities related from parents. With six months of age, she was submitted to a medical evaluation which showed a normal neurological result. MRI of the lumbosacral region was requested and identified an appendage lesion with approximately 2 cm, with no communication with the spinal cord, which developed a false human tail (Figures 1(a) and 1(b)). The surgical resection began with a half-moon incision, made in the lumbosacral region, 0.5 cm above the lesion. Opening plans following the fibroelastic tissue found, until its communications with the dura. Opening the dura for investigation of possible intradural component and surgical revision with microscopy (using microsurgical technique). Fibroelastic tissue was identified and no structure suggestive of nerve tissue within the lesion was found, confirmed by the histopathological study. A complete resection of the lesion was made.

## 3. Case 2

**Figure 2:**
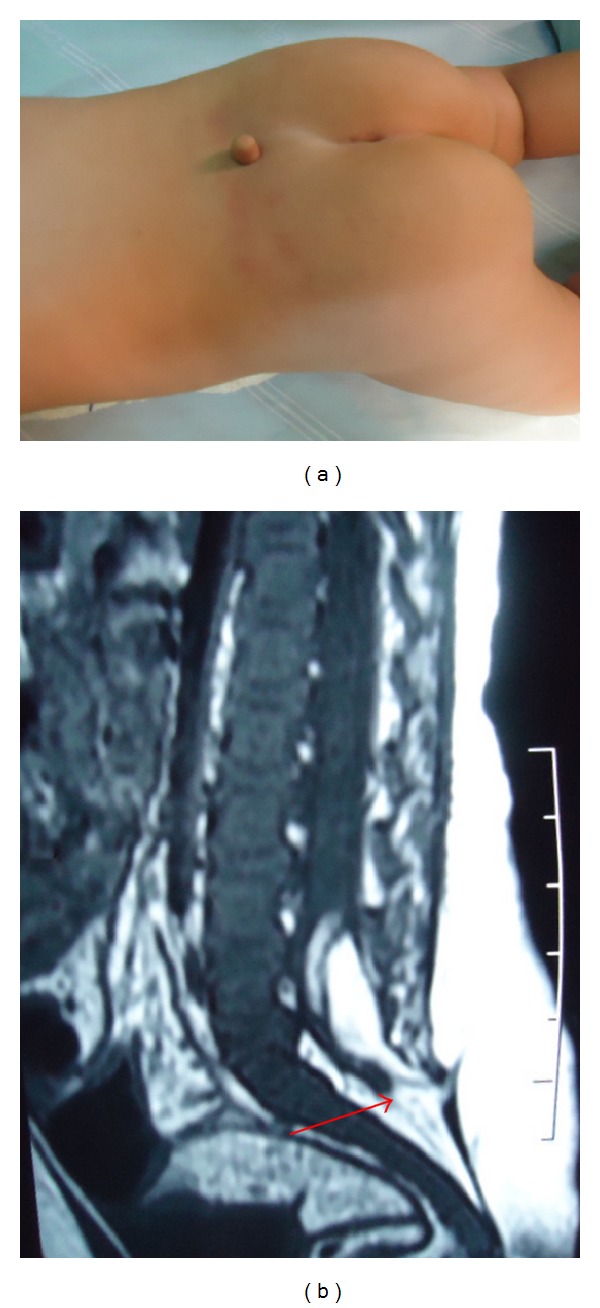
(a) Lesion at the lumbosacral region, type tail. (b) Confirmation by a lumbosacral MRI that showed a spinal dysraphism associated with intradural lipoma.

Male child, with 8 months old, showed a lesion in the lumbosacral region, type tail. Normal neurological exams and lumbosacral MRI were performed and confirmed spinal dysraphism associated with intradural lipoma (Figures 2(a) and 2(b)). In this case, after opening the dura—using microsurgical technique—partial resection of the intradural lipoma associated with the human tail was performed. 

## 4. Case 3

**Figure 3:**
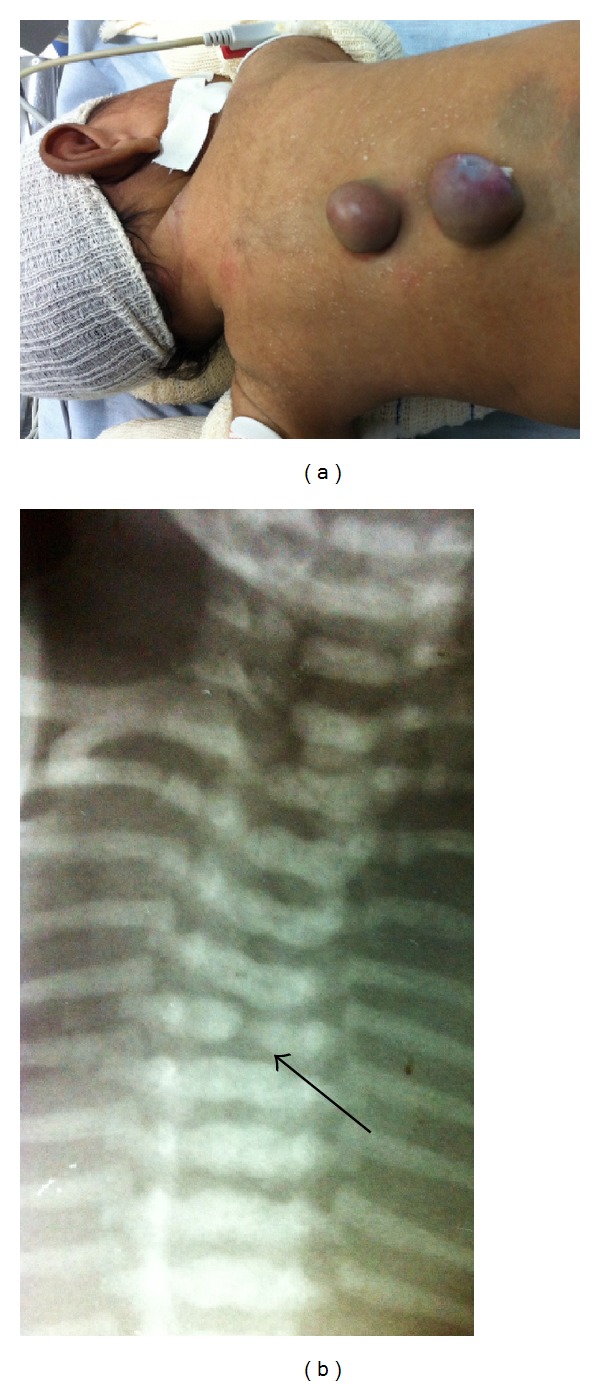
(a) A newborn with multiple thoracic myelomeningocele. (b) Plain radiograph of the chest, showing a reduction and a fissure in the body of the fifith thoracic vertebrae.

A neonate (18 days of life) with two intact cystic lesions was detected in the posterior thoracic region, of 2 cm (the superior) and 3 cm (the inferior) (Figure 3(a)). Concerning prenatal followup, the mother started to use folic acid after the third month of pregnancy and had no familiar history of MM. The clinical diagnosis was confirmed by the ultrasonography (US) of the lesions, confirming that it was two thoracic MMs. A chest radiography exam showed, at the level of the fifth thoracic vertebrae, a reduction of the left part of the vertebra, as well as a fissure (Figure 3(b)). Surgery was performed to correction and closure of both MMs, even before the MRI, because of the risk of rupture of lesions and consequent exposure of the neural tissue, increasing the chance of an infection. Classical surgery for correction of both MMs was performed. During hospitalization, a daily measure of head circumference was performed, as well as transcranial US in each three days, to monitor the size of ventricles. Five days after surgery, it was verified that the neonate had large ventricles and bulging fontanels (hydrocephalus), and a ventricular peritoneal shunt was placed. 

As a routine, our unit provides guidance to the patient families, for outpatient and multidisciplinary accompanying, in cases of spinal dysraphism (pediatric neurosurgery, pediatric neurology, orthopedics, urology, and pediatric physical therapy) and were submitted to MRI examination, in search of other neurological congenital malformations. 

## 5. Discussion 

At the 6th week of pregnancy, the human embryo has a conspicuous tail protruding from the trunk that has 10 to 12 caudal vertebrae. Between the 7th and 8th week of embryogenesis, the tail regresses as the vertebrate portion retracts into the soft tissues. By the end of the 8th week, the tail has completely disappeared. It is generally believed that the vestigial human tail probably arises from the distal invertebrated portion of the embryonic tail found at this stage of pregnancy [5]. Human tails are classified as true tails (persistent vestigial) and pseudotails. The true tail contains adipose and connective tissue, striated muscle, blood vessels, and nerves covered by normal skin with the usual number of hair follicles and sweat glands, but boneless, with no cartilage, notochord, or spinal cord elements. Tail movements has been described in some cases [5–7]. Both cases described here were—probably—pseudotails, since only the fibroelastic tissue was identified. Pseudotails include also other forms of caudal appendages such as an elongated coccyx, teratoma, lipoma, parasitic fetus, fibrolipoma, chondromegalic protrusion, and prolonged sacrum [8, 9]. 

There are only a few cases reporting human tail in the literature. It is considered to be a marker of underlying intraspinal pathology of occult spinal dysraphism [2]. A multitude of spinal cord and spine anomalies associations, including spina bifida, lipomeningoceles, myelomeningocele, intraspinal lipoma, spinal cord tethering, and coccygeal vertebrae, were described in patients with human tail [10, 11]. The caudal appendage that mostly occurs with occult spina bifida or spinal dysraphism is pseudotails [2], as described above. Performing the MRI is crucial to identify intradural extension, and when present, microsurgical resection and opening the dura are essential. 

Concerning multiple MMs, in one of the largest series of multiple dysraphisms described in the literature, for a total of 10 cases, only 3 patients were reported with multiple MMs [4]. Most commonly in cases of multiple spinal dysraphism, MMs are associated with lipomas, spina bifida, and tight *filum terminale*, with the occurrence of multiple MMs in the same patient being rare [4, 12]. In a previous study, made in our Pediatric Neurosurgical Unit, addressing congenital diseases of the central nervous system, it was observed that—though they were oriented about the need for prenatal care and about the regular use of folic acid—a large number of pregnant women did not use it regularly [13], which increases the risk of neural tube imperfections [14–16]. We recommended, as others, that at least 400 *μ*g of a folic acid supplement must to be consumed daily. In case of intermediate to high risk for neural tube defects (such as previous history of neural tube defects), 4 to 5 mg of folic acid should be taken [16]. 

## 6. Conclusions

Human tails, as well as multiple dysraphism, are very uncommon congenital spinal malformations. MRI is the method of choice to diagnose spinal dysraphic lesions, and whole spinal MRI is required in search of other occult dysraphisms in cases of multiple MMs. In cases of human tails, we consider that the opening of dura is imperative, if MRI shows intradural abnormalities, to avoid future tethered cord syndrome. Fortunately microsurgical techniques could be done without complications in both spinal malformations.
